# Activity of the antioxidant enzyme paraoxonase in Argentinean children living at high altitude

**DOI:** 10.1080/13510002.2017.1370783

**Published:** 2017-08-30

**Authors:** V. Hirschler, M. Martín, K. Oestreicher, C. Molinari, W. Tetzlaff, E. Botta, L. Boero, F. Brites, on Behalf of San Antonio de los Cobres Study Group

**Affiliations:** a University of Buenos Aires, Buenos Aires, Argentina; b Laboratory of Lipids and Atherosclerosis, Department of Clinical Biochemistry, School of Pharmacy and Biochemistry, University of Buenos Aires-CONICET, Buenos Aires, Argentina

**Keywords:** High altitude, indigenous school children, PON1, HDL, atherosclerosis

## Abstract

**Background:** Children living at high altitude in San Antonio de los Cobres (SAC), Argentina, were shown to have lower high-density lipoprotein cholesterol (HDL-C) levels than Buenos Aires (BA) children. HDL antioxidant capacity is mainly attributed to paraoxonase1 (PON1).

**Objective:** To compare PON1 activity in indigenous SAC vs. BA children.

**Methods:** A cross-sectional study compared 158 SAC vs. 97 BA children (6–16 years). Anthropometric data and lipoprotein profile were measured. PON1 was evaluated employing paraoxon (PON) and phenylacetate (ARE) activity.

**Results:** The prevalence of overweight/obesity was lower in SAC than in BA children (18.3 vs*.* 30.9%). Triglycerides (1.34 vs. 0.90 mmol/l), apo B (0.84 vs*.*0.72 g/l), apo A-I (1.33 vs*.* 1.27 g/l), and ARE activity (100 vs*.* 90 µmol/ml/min) were higher, while HDL-C (1.16 vs*.* 1.32 mmol/l) and PON activity (170 vs. 203 nmol/ml/min) were lower in SAC than in BA. Separate multiple linear regression analyses showed that SAC children had significantly higher triglyceride (Beta −0.38), apo B (Beta −0.34), and ARE (Beta −0.36) plus lower HDL-C (Beta 0.33) and PON (Beta 0.25) compared with BA; adjusted for age, gender, and BMI.

**Conclusion:** SAC showed an unfavorable lipoprotein profile, lower PON and higher ARE activities compared with BA children, suggesting the presence of altered HDL metabolism and antioxidant capacity.

## Introduction

Abnormal lipid levels can emerge during childhood and adolescence and persist into adulthood [1]. Thus, their early detection is important when taking into account that dyslipidemia is a modifiable risk factor for cardiovascular disease. Furthermore, depending on its etiology, it can be corrected by healthy lifestyle and, if necessary, by timely medical intervention. Several cases of dyslipidemia diagnosed in early childhood are of genetic origin, though the causality of other factors should not be discarded.

Past studies have compared the prevalence of dyslipidemia between urban and rural children [2,3], while data from children living at high altitude are scarcer and have mainly focused on the presence of conditions such as stunted growth [4], cognitive dysfunction [5] and respiratory infections [6]. A previous study, carried out in indigenous Argentinean school children living at 3750 m above sea level in San Antonio de los Cobres (SAC), Salta, showed a higher prevalence of high triglyceride and low high-density lipoprotein cholesterol (HDL-C) levels compared with BA children, suggesting that high altitude or ethnicity could be associated with dyslipidemia [7]. Furthermore, different studies have characterized lipoprotein profile in adults who resided in those environments [8,9]. Among them, increased prevalence of hypertriglyceridemia and low HDL-C levels has also been reported in Peruvian adults who lived at 4100 m [8]. Likewise, Santos et al. [9] showed a prevalence of hypercholesterolemia of 37% and low HDL-C levels of 25% in Northern Chile (2000–4500 m).

It has been reported that the major high altitude populations live on the Andean, Tibetan, and east African plateau, where they experience the unique stress of hypobaric hypoxia [10]. Barometric pressure falls with increasing altitude, which triggers several physiological adaptations in people who live there [10]. Bailey et al. [11] showed that people exposed to high altitude would be at an increased risk of oxidative stress, which has been associated with numerous diseases, including atherosclerosis. In addition, factors other than hypoxia may contribute to the generation of free radicals, including extreme temperature and ionizing radiation [12].

HDL is the only antiatherogenic lipoprotein and, among different functions, it protects low density lipoprotein (LDL) against oxidative modification [13]. This event is believed to be central for the initiation and progression of atherosclerosis. HDL antioxidant activity is mainly attributed to the enzyme paraoxonase (PON) 1, which has been proved to prevent lipid-peroxide accumulation on LDL [14]. PON1 is a glycoprotein mainly secreted by the liver and in plasma it circulates bound to HDL particles. PON1 activity may be evaluated employing two different substrates, paraoxon (PON activity) and phenylacetate (arylesterase, ARE, activity). Both measurements are complementary given that PON1 activity better reflects the enzyme antioxidant activity, while the ARE activity represents an estimate of its concentration [15].

We hypothesize that, as a result of the exposure to high altitude, SAC children would display a more atherogenic lipid profile, in addition to alterations in HDL composition and paraoxonase activity.

We are unaware of any previous research that explored lipoprotein metabolism beyond the measurement of lipid concentrations in children exposed to high altitude. Therefore, the objective of this study was to compare PON1 activity in indigenous SAC vs. urban Buenos Aires (BA) children.

## Methods

### Subjects

A school from SAC was randomly selected in October 2015. Ninety-eight percent of the SAC population was indigenous. Exclusion criteria were: (1) missing anthropometric, blood pressure, or biochemical data; (2) not fasting for at least 12 hours; (3) the presence of diabetes or other chronic diseases; (4) the use of medication that could affect blood pressure, glucose, or lipid metabolism; (5) the informed consent form not being signed; and (6) the child’s refusal to participate. The study was approved by the Human Rights Committee of the Salta Health Ministry. Each parent gave written informed consent and each child assented to participate after an explanation of the study and before its initiation. A school from BA suburbs was randomly selected in November 2015. The BA children belonged to a mixed population composed of approximately 85% of European descent (largely Spanish and Italian), with the remainder of mixed European and American Indian (12%) or American Indian (3%) descent [16]. A cross-sectional study was performed between October and November 2015.

### Clinical characteristics

Height and weight were measured with the subject wearing lightweight clothing and without shoes. Waist circumference was measured with a flexible steel tape with the subject standing; the anatomic landmark was midway between the lowest portion of the rib cage and the iliac crest. BMI was calculated as weight (kg) divided by height^2^ (m^2^). Overweight and obesity were defined as BMI 85th to <95th and ≥95th percentiles, respectively, according to age and gender. Blood pressure was measured according to the Seventh Report of the Joint National Committee on Prevention, Detection, Evaluation, and Treatment of High Blood Pressure.

### Study protocol and samples

Blood samples were obtained from the antecubital vein of each participant after a 12-hour overnight fast. Serum was immediately isolated by low speed centrifugation and stored at −80°C before its use for the determination of glucose, lipids, apo A-I, and apo B concentrations in addition to PON1 activities.

### General biochemical determinations

Plasma levels of glucose, total cholesterol, triglycerides, and HDL-C were measured by standardized methods in a COBAS^®^ C501 autoanalyser (Roche, Mannheim, Germany). LDL-C was calculated as the difference between TC and the cholesterol contained in the supernatant obtained after selective precipitation with polyvinyl sulfate. Plasma apo A-I and apo B levels were quantitated by immunoturbidimetry (Roche, Mannheim, Germany). Abnormal lipid levels were defined according to the National Institutes of Health’s Expert Panel on Integrated Guidelines for Cardiovascular Health and Risk Reduction in Children and Adolescents.

### Measurement of PON1 activities

PON1 activity was evaluated employing two different substrates, paraoxon (PON activity) and phenylacetate (ARE activity) (Sigma Chemical Co, St. Louis, MO, U.S.A.) [17]. PON 1 activity was assessed by adding serum samples (20 μl) to 2 ml Tris/HCl 10 buffer (100 mmol/l, pH = 8.0) containing 2 mmol/l CaCl_2_, 2.6 mmol/l paraoxon (O,O-diethyl-O-*p*-nitrophenylphosphate), and 1.0 mol/l NaCl. The rate of generation of *p*-nitrophenol was determined at 405 nm and 25°C, in a Hitachi U-1100 spectrophotometer. Increases in the absorbance were recorded at 45-second intervals during 5 minutes, after 30 seconds of initial pre-incubation. Enzymatic activity was calculated from the molar extinction coefficient (17,000 mol^−1 ^l cm^−1^) and results were expressed as nmol/ml min. ARE activity was measured by adding serum samples (20 μl of 1/20 dilution in distilled water) to 2 ml Tris/acetate buffer (50 mmol/l, pH = 7.8) containing 20 mmol/l CaCl_2_ and 4.4 mmol/l phenylacetate. The rate of generation of phenol was determined at 270 nm and 25°C, in a Hitachi U-1100 spectrophotometer. Increases in the absorbance were recorded at 45-second intervals during 5 minutes, after 30 seconds of initial pre-incubation. Blanks were included to correct for the spontaneous hydrolysis of phenylacetate. Enzymatic activity was calculated from the molar extinction coefficient (1310 mol^−1 ^l cm^−1^) and results were expressed as μmol/ml.min. PON phenotypes were determined by the double substrate method [15].

Measurements were all carried out within the same assay. Within-run precision was 4.6% for PON activity and 4.2% for ARE activity.

### Data analysis

Descriptive statistics for raw variables were presented as means ± standard deviations. When comparing two groups with normally distributed data, a student *t*-test was performed. Bonferroni’s adjustment was carried out when many comparisons were performed. When the homogeneity of the variances could not be proved, the Brown–Forsythe test was used. Variables with an asymmetric distribution were logarithmically transformed for analyses. The primary focus of the analysis was to compare PON1 activity in indigenous SAC vs. urban children from BA. Several multiple regression analyses were performed using triglycerides, HDL-C, apo B levels, and PON1 activity as dependent variables and age, gender, BMI, and location (SAC vs. BA) as independent variables. All tests were 2-sided and *P*-values of less than 0.05 were considered statistically significant. Analyses were performed using the statistical software package SPSS version 22.0 (Chicago, IL, U.S.A.).

### Compliance with ethical standards

The current study was performed in accordance with the Code of Ethics of the World Medical Association (Declaration of Helsinki) for experiments involving humans and the Uniform Requirements for manuscripts submitted to Biomedical Journals published by the International Committee of Medical Journal Editors. The authors had full access to the data and take responsibility for its integrity.

## Results

**Figure 1.**
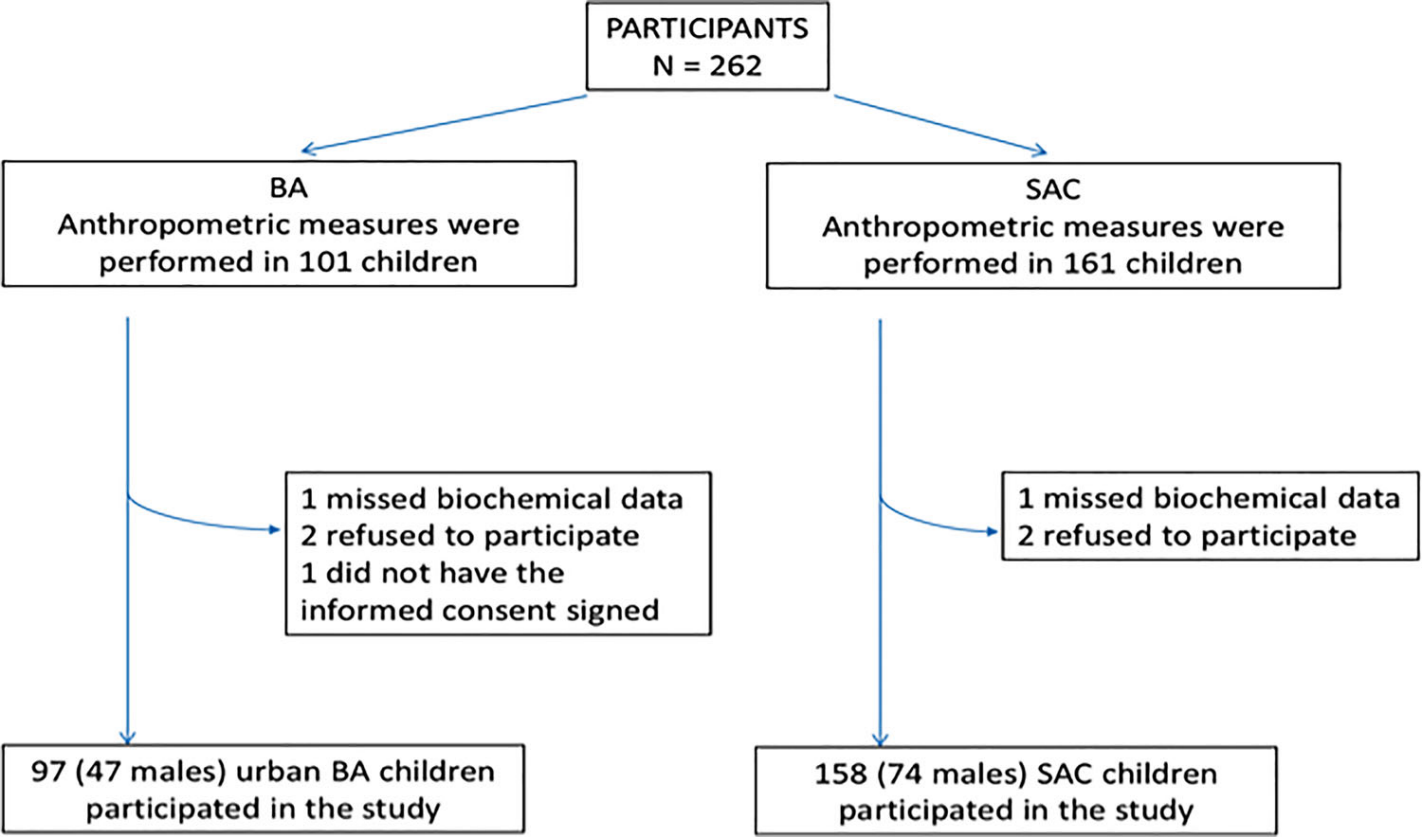
Study flowchart. BA: Buenos Aires; SAC: San Antonio de los Cobres.

Figure 1 shows the study flowchart. One hundred and fifty-eight (74 males) SAC children were compared with 97 (47 males) BA children (6–16 years). SAC children presented significantly lower BMI. The prevalence of overweight/obesity was significantly lower in SAC (26/158; 18.3%) than in BA (30/97; 30.9%) (*P* < 0.01). There was not a significant difference in the prevalence of overweight/obesity between genders in both communities. Regarding the lipoprotein profile, SAC children showed significantly higher triglyceride and apo B levels, while HDL-C levels were significantly lower compared with BA children. Surprisingly, apo A-I concentration was also higher in SAC than in BA group (Table 1). Accordingly, the prevalence of high triglycerides (28/158; 17.7% vs*.* 4/97; 4.1%; *P* < 0.01) and low HDL-C (14/158; 8.9% vs*.* 4/97; 4.1%; *P* < 0.01) was significantly higher in SAC than in BA. There was not a significant difference in the prevalence of high triglycerides or low HDL-C between genders in both communities.

**Table 1.** Clinical and metabolic characteristics in SAC and BA children.

	SAC children (*n *= 158)	BA children (*n *= 97)
Age (years)	10.5 ± 2.0	9.2 ± 2.1**
BMI (kg/m^2^)	18.3 ± 2.8	19.1 ± 4.7**
Z-BMI	0.3 ± 0.9	0.6 ± 1.1*
Z-Height	−1.3 ± 0.9	−0.4 ± 1.4*
Systolic BP (kPa)	16.26 ± 1.86	12.13 ± 1.73**
TG (mmol/l)	1.34 ± 0.64	0.90 ± 0.44**
TC (mmol/l)	4.04 ± 0.59	4.09 ± 0.75
HDL-C (mmol/l)	1.16 ± 0.21	1.32 ± 0.26**
LDL-C (mmol/l)	2.43 ± 0.57	2.54 ± 0.70
Apo A-I (g/l)	1.33 ± 0.17	1.27 ± 0.19**
Apo B (g/l)	0.84 ± 0.18	0.72 ± 0.19**
TC/HDL-C	3.5 ± 0.8	3.1 ± 0.7**
LDL-C/HDL-C	2.1 ± 0.6	1.9 ± 0.6*
Apo A-I/Apo B	1.7 ± 0.5	1.9 ± 0.5*
LDL-C/Apo B	1.1 ± 0.1	1.4 ± 0.2**
TG/HDL-C	2.8 ± 1.9	1.6 ± 0.8**
HDL-C/Apo A-I	0.3 ± 0.1	0.4 ± 0.1**

SAC: San Antonio de los Cobres; BA: Buenos Aires; BMI: body mass index; BP: blood pressure; TG: triglycerides; TC: total cholesterol; HDL-C: high-density lipoprotein cholesterol; LDL-C: low density lipoprotein cholesterol; Apo: apolipoprotein. Data are presented as mean ± SD. Z-score is a quantitative measure of the deviation of a specific variable taken from the mean of that population. CDC z-BMI takes into account age and gender. Significance: *P < 0.05 and **P < 0.01.

Different lipid-related indexes were calculated. Interestingly, total cholesterol/HDL-C, LDL-C/HDL-C, apo B/apo A-I, and triglycerides/HDL-C ratios were significantly higher, while LDL-C/apo B and HDL-C/apo A-I were significantly lower in SAC than in BA children.

**Figure 2.**
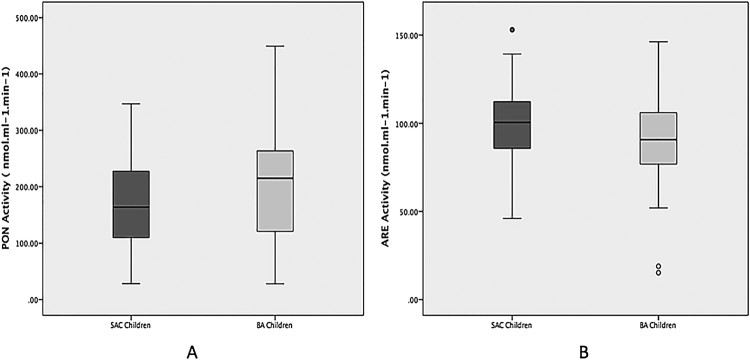
PON (A) and ARE (B) activities from SAC and BA children. The boxes define the 25th and 75th percentiles, and enclose the median; the extensions define the range of values. PON: paraoxonase; ARE: arylesterase; SAC: San Antonio de los Cobres; BA: Buenos Aires. *P < 0.05 vs. SAC children.

Evaluations of PON1 enzyme were carried out by means of its PON and ARE activities. Given that PON activity is partially determined by the R/Q polymorphism, the phenotypic distribution was evaluated in both groups. This analysis showed similar distribution of the QQ, QR, and RR phenotypes between SAC (3, 95, 2%; respectively) and BA (2, 95, 3%; respectively) children (*χ*^2^ = 0.44; *P* = 0.80). This similarity allowed the comparison of PON activity between both groups. Then, PON activity was significantly lower in SAC than in BA children, whereas ARE activity was significantly higher in SAC than in BA children (Figure 2). Accordingly, PON/ARE ratio, which could be considered a surrogate of the enzymatic specific activity, was significantly reduced in SAC compared with BA children (1.7 vs. 2.2, respectively; *P* < 0.01).

Separate multiple linear regression analyses showed that SAC children had higher triglyceride, apo A-I and apo B, and lower HDL-C levels than BA children; adjusted for age, gender, and BMI. Furthermore, we found lower PON and higher ARE activities in SAC than in BA children; adjusted for gender, age, and BMI (Table 2).

**Table 2.** Multiple linear regression analyses of data from SAC and BA children.

Dependent variable	Unstandardized coefficients		Standardized coefficients	*t*	*P*
	B	Std. error	Beta		
TG	−43.81	7.25	−0.38	0.5	<0.01
HDL-C	6.58	1.24	0.33	5.24	<0.01
Apo A-I	−5.59	2.58	−0.15	−2.16	0.03
Apo B	−13.82	2.56	−0.34	−5.17	<0.01
PON	44.74	12.14	0.25	3.68	<0.01
ARE	−9.71	2.89	−0.23	−3.36	<0.01

Separate multiple linear regression models, adjusted for gender, age, and BMI. SAC: San Antonio de los Cobres; BA: Buenos Aires; TG: triglycerides, HDL-C: high-density lipoprotein cholesterol; Apo: apolipoprotein; PON: paraoxonase; ARE: arylesterase.

Even though there were no differences in phenotype distribution between the two populations, we still decided to perform a stratified analysis to test if the differences observed in the comparison between whole populations could be attributed to a particular phenotype. Indeed, all differences found in the analysis for complete populations were replicated in the comparison between AB individuals (Table 3). This was to be expected, given that, as previously stated, the majority of the individuals in both population were heterozygous. We found no statistically significant differences when comparing either AA or BB individuals.

**Table 3.** Multiple linear regression analyses of data from SAC and BA children of the AB phenotype.

Dependent variable	Unstandardized coefficients		Standardized coefficients	*t*	*P*
	B	Std. error	Beta		
TG	−45.53	7.07	−0.41	−6.43	<0.01
HDL-C	6.92	1.29	0.35	5.35	<0.01
Apo A-I	−5.23	2.60	−0.14	−2.01	0.04
Apo B	−11.24	2.68	−0.29	−4.19	<0.01
PON	43.08	12.21	0.25	3.53	<0.01
ARE	−5.31	2.85	−0.14	−1.86	<0.01

Separate multiple linear regression models, adjusted for gender, age, and BMI. SAC: San Antonio de los Cobres; BA: Buenos Aires; TG: triglycerides, HDL-C: high-density lipoprotein cholesterol; Apo: apolipoprotein; PON: paraoxonase; ARE: arylesterase.

## Discussion

Our results show that SAC children had lower prevalence of overweight obesity than BA children. In addition, they had lower PON and higher ARE activities than BA children, adjusted for age, gender, and BMI. It should be noted that the comparison of SAC and BA children of the AB phenotype showed significant differences in all the same parameters as the comparison for complete populations. Even though we found no differences in the comparison between AA and BB children, this could be attributed to the low prevalence of both homocigous phenotypes observed in our study. Given that ARE better reflects the enzyme concentration and PON its activity, these results would specifically suggest a deteriorated antioxidant capacity, one of the main HDL antiatherogenic properties. A previous prospective study showed that low PON activity was a predictive risk factor for subsequent coronary events independent of all other established risk factors, with the exception of HDL-C [18]. As far as we know, no information is available on the altered PON1 activity in children living at high altitude. Furthermore, there is no information about HDL antioxidant capacity in pediatric populations living at high altitude. Interest in PON1 is mainly due to the knowledge that this enzyme protects LDL and HDL from oxidative stress [14,19].

The prevalence of overweight/obesity was significantly lower in SAC than in BA children. There are several plausible mechanisms relating altitude and obesity, including hypoxia, mean annual temperature, physical activity, leptin signaling, metabolic demands, and ethnicity. Previous literature has suggested that reduced temperature at increased elevation may lead to weight loss through catabolic effects [20]. Consistently, SAC is located in the mountains at 3750 m above sea level with a mean annual temperature of 7.7°C and a wind speed of 21 km/hour [16]. Therefore, despite the sunny weather, SAC children must endure a cold and windy climate. This could be the reason for the low prevalence of obesity in SAC children. Another possible mechanism that may contribute to the lower prevalence of obesity in SAC could be the differences in energy expenditure in physical activity. Because SAC is located in mountainous terrain, children in SAC may expend more energy than BA children, because they need to walk uphill on a regular basis. In contrast, BA children may expend less energy even if they walk the same distance every day. Finally, exposure to hypoxia has been shown to stimulate hypoxia inducible factor 1, which appears to be an important regulator for the expression of the leptin gene – a hormone secreted by adipose tissue that produces negative feedback on appetite – and inversely associated with obesity [21]. Consistently, this study showed that the prevalence of overweight and obesity was approximately four-fold lower in SAC than in CH children. In summary, this finding presents a striking example of the variation in the prevalence of obesity found in these communities.

High risk of cardiovascular disease would not only derive from altered PON1 activity but also from the presence of a more atherogenic lipoprotein profile in SAC children. In fact, SAC children showed higher triglyceride and apo B levels, and lower HDL-C concentration than BA children. Moreover, high atherogenic risk is also supported by higher total cholesterol/HDL-C and LDL-C/HDL-C and lower apoA-I/apoB ratios [22]. Besides, triglycerides/HDL-C index was also higher in the SAC group, suggesting the presence of insulin resistance. Furthermore, higher levels of triglycerides/HDL-C index were associated with increased proportion of small and dense LDL particles [23,24], with the latter also being confirmed by a reduced LDL-C/apo B ratio [25]. It is interesting to note that the above-mentioned abnormalities in the lipoprotein profile were present even though the prevalence of obesity was lower in SAC than in BA children, thus highlighting the concurrence of other conditioning factors such as oxidative stress.

Exposure to high altitude has been associated with increased lipid peroxidation [11,26]. Therefore, SAC children evaluated in the present study, who live at 3750 m above sea level and are chronically exposed to hypoxia, could be permanently undergoing a condition of high oxidative stress. Sources of oxidative stress in altitude may include exposure to ultraviolet light and low temperatures, increased exercise, dietary factors, and stimulation of the sympathetic nervous system [12]. Previous studies of populations chronically living at high altitude (4300 m) in the Andes showed elevated levels of lipid peroxidation products, measured as plasma levels of thiobarbituric acid reactive substances and urinary concentration of 8-iso Prostaglandin F2α [26], which may impair antioxidant defenses [27]. In fact, oxidative stress has been observed under acute, chronic, and chronically intermittent exposure to high altitude in either animal models or humans [26,28–32]. Under conditions of increased oxidative stress, PON1 is known to suffer oxidative modification of its structure, which leads to a loss of its functionality [33]. Thus, in SAC children, low PON activity would seem to be the consequence of increased oxidative stress. However, reduction of PON activity in SAC children might also be conditioned by other factors such as higher triglyceride levels compared with BA children. A previous study performed by our group showed that primary hypertriglyceridemia was associated with low PON antioxidant activity [17].

In contrast, ARE activity resulted to be higher in SAC than in BA children. Exclusive reduction in PON activity has been described in different pathologies and conditions such as cardiovascular disease [18] and metabolic syndrome [34], in which it is frequent to detect low PON and unaltered ARE activities. Interestingly, increased ARE activity in SAC children could be in direct relation to higher apo A-I levels found in this population in comparison with BA children. It has been previously reported that apo A-I concentration was significantly increased in healthy Chinese adults who lived at 3760 m above sea level [35]. Accordingly, studies performed in animal models showed an increment in apo A-I levels in rats susceptible to hypobaric hypoxia [36]. Apo A-I is the main protein component of HDL particles and it is considered a surrogate of particle number [37]. So, as HDL is the unique carrier of PON1, it is expected that variations in HDL particle number would lead to parallel variations in PON1 concentration. Nevertheless, it is important to note that PON1 concentration is better reflected by measurement of ARE than by determination of PON activity, which is influenced by R/Q polymorphism and clearly reflects the enzyme intrinsic activity [15].

In this frame characterized by the presence of a high number of HDL particles with reduced cholesterol content, low PON antioxidant activity and the presence of oxidative stress, it is expected to find dysfunctional HDL particles, which would in turn be defective in their capacity to promote cellular cholesterol efflux. Impairment of this antiatherogenic pathway induced by oxidative stress has been extensively reported [38]. Therefore, oxidative stress-induced decrease in cellular cholesterol efflux could help to explain the lower HDL-C levels found in SAC children compared with BA children.

Different limitations of this study should be acknowledged. First, it was a cross-sectional analysis, and thus, the directionality of the associations cannot be established. Second, we were unable to evaluate the association between PON1 activity and pubertal development stage. Third, this study lacked information regarding family history of CVD, pubertal status, physical activity, and diet history, all of which are known to influence CVD. Despite these limitations, appropriate analysis of cross-sectional data represents a useful initial step in identifying lower PON1 activity in children living at high altitude compared with those at sea level, adjusted for several confounding variables. Moreover, our study has several strengths that should be mentioned. First, this study is one of very few that examined PON1 activity in relation to various metabolic outcomes of interest in school children from different communities. Finally, most studies in children included only children who were overweight or obese, while this study included apparently healthy school children.

## Conclusion

This study shows that SAC children living at high altitude, a condition known to be characterized by high oxidative stress, showed a more atherogenic lipoprotein profile, which certainly contributed to determine a lower PON1 antioxidant activity compared with BA children. Their associations with increased ARE activity, higher apo A-I levels, and decreased HDL-C concentration would reflect abnormal HDL metabolism and functionality. Overall, these findings are strongly suggestive of higher risk of earlier cardiovascular disease in this community of indigenous children living at high altitude. Additional longitudinal studies should be performed to confirm these findings.

## Geolocation information

This study was carried out in the cities of Buenos Aires (34 35′59″S, 58°22′55″W), Argentina, and San Antonio de los Cobres (24°13′32″S, 66°19′9″W), Salta, Argentina.
